# Influence of isoflurane exposure in pregnant rats on the learning and memory of offsprings

**DOI:** 10.1186/s12871-018-0471-2

**Published:** 2018-01-11

**Authors:** Wei Huang, Yunxia Dong, Guangyi Zhao, Yuan Wang, Jingjing Jiang, Ping Zhao

**Affiliations:** Department of Anesthesiology, Shengjing Hospital of China Medical University, No. 36 SanHao Street, HePing District, ShenYang, Liaoning Province People’s Republic of China

**Keywords:** Isoflurane, CREB, Pregnancy, Memory, Offsprings

## Abstract

**Background:**

About 2% of pregnant women receive non-obstetric surgery under general anesthesia each year. During pregnancy, general anesthetics may affect brain development of the fetus. This study aimed to investigate safe dosage range of isoflurane.

**Methods:**

Forty-eight SpragueDawley (SD) pregnant rats were randomly divided into 3 groups and inhaled 1.3% isoflurane (the Iso1 group), 2.0% isoflurane (the Iso2 group) and 50% O_2_ alone (the control group) for 3 h, respectively. Their offsprings were subjected to Morris water maze at day 28 and day 90 after birth to evaluate learning and memory. The expression of cAMP-response element binding protein (CREB) and phosphorylated cAMP-response element binding protein (p-CREB) was detected in the hippocampus dentate gyrus.

**Results:**

Less offsprings of Iso2 group were able to cross the platform than that of the control group (*P* < 0.05). Accordingly, the Iso2 offsprings expressed p-CREB mainly in the subgranular zone in contrast to the whole granular cell layer of hippocampus dentate gyrus as detected in the Iso1 and control offsprings; the expression level of pCREB was also lower in the Iso2 than Iso1 or control offsprings (*P* < 0.05).

**Conclusion:**

Inhalation of isoflurane at 1.3% during pregnancy has no significant influence on learning and memory of the offspring; exposure to isoflurane at 2.0% causes damage to spatial memory associated with inhibition of CREB phosphorylation in the granular cell layer of hippocampus dentate gyrus.

## Background

Approximately, more than 2% of pregnant women receive non-obstetric surgery under general anesthesia [1, 2]. In humans, brain development mainly occurs in the fetal period when the proliferation, differentiation and migration of neurons and the formation and modification of synapses as well as myelin are very active. Thus, during that time, the fetal development of central nervous system is extremely vulnerable to both internal and external environmental changes and neurons without formation of synapses will become apoptotic [3, 4]. General anesthesia during pregnancy may affect brain development of the fetus and their learning abilities.

However, there is no guideline for isoflurane usage during pregnancy due to lack of clinical studies [5]. In 1985, Uemura et al. first found that the fetus exposed to halothane affected synaptic development in the neonatal brains [6], which have confirmed by increasing evidence [7, 8]. It was proposed that anesthetics used in general anesthesia increase the apoptosis of immature neurons, causing damage to the nervous system in fetus [9]. To date, a variety of studies have shown that high concentration of anesthetics in general anesthesia cause damages to nervous system, but the influence of these anesthetics at a clinical or subclinical concentration on fetal brain development is unclear.

Therefore, it is important to investigate the influence of general anesthesia on brain development of offspring in order to guide anesthesia in pregnant women receiving non-obstetric surgery. In the present study, pregnant rats were exposed to isoflurane at different concentrations and subjected their offsprings to the behavior study, aiming to investigate the influence of isoflurane exposure during pregnancy on the memory and learning abilities of the offspring as well as to explore the range of safe doses, which may provide evidence for the clinical use and investigations of anesthetics. We hypothesize that isoflurane inhalation during pregnancy compromises the offspring’s learning abilities and memory in a concentration-dependent manner.

## Methods

This study was approved by the Ethics Committee of Affiliated Shengjing Hospital of China Medical University, and specific pathogen free SD pregnant rats weighing 380–420 g were purchased from the Experimental Animal Center of Affiliated Shengjing Hospital of China Medical University. Animals were housed at 22–24 °C, 40–60% humidity with a 12-h light /dark cycle and had free access to food and water. Rats at the gestational age of 21 days (E21) were used in subsequent experiments. According to the isoflurane dose, rats were divided into 3 groups: the Iso1 group (1.3% isoflurane), the Iso2 group (2.0% isoflurane) and the control group (0% isoflurane; O_2_).

In the absence of anesthesia, intratracheal intubation was difficult in the control group. Thus, all the rats retained spontaneous breathing and did not receive intratracheal intubation. Inhalation of isoflurane at a high concentration may inhibit respiration and cause hypoxia. Thus, in our pilot study, pregnant rats at the gestational age of 20 days (E20) were anesthetized intraperitoneally with pentobarbital sodium and catheter indwelling was done in the right carotid artery; rats were then allowed to recover at room temperature. At E21, rats were placed in a box filled with prefilled gas according to the following groups: 50% O_2_ was administered in the control group; 1.3% isoflurane was administered in the Iso1 group (50% oxygen, balanced with nitrogen); 2.0% isoflurane was administered in the Iso2 group (50% oxygen, balanced with nitrogen). All rats were retained spontaneous breathing and exposed in the box for 3 h (the concentrations of isoflurane and oxygen were monitored). The mean arterial blood pressure was continuously monitored via a catheter in the carotid artery, and arterial gas analysis was performed hourly. The results showed that inhalation of isoflurane at 1.3% or 2.0% had no influence on the arterial gas and mean arterial blood pressure. Rats used in pilot study will not be used for formal study.

In this study, a total of 48 rats at E21 were randomly assigned into 3 groups and exposed to isoflurane at the predesigned concentration for 3 h. Animals were allowed to recover at room temperature and housed until they delivered. The number of fetuses was recorded, and healthy male neonatal rats were used in the experiments. At day 28 after birth (P28), the male offsprings were randomly assigned into two groups: one for Morris water maze (MWM) test to evaluate memory and learning and the other one were housed until day 90 after birth (P90) to receive the same MWM test.

MWM test used a round swimming pool sized 150 cm in diameter and 60 cm in height with a platform sized 10 cm in diameter in the maze. The removable platform was 1.5 cm lower than the water surface. The visual cues (a variety of figures) on the maze’s inner wall remained unchanged during the study. Training and examination were performed in the water at 20 °C. After each examination, rats were dried under a lamp and returned to the cages.

Place navigation test was performed for consecutive 5 days. In brief, platform were placed in a quadrant (the 4th quadrant in this study). At predesigned time point, rats were placed in a random quadrant (once for each quadrant). If the rat found the platform within 90s, it was allowed to stay on the platform for 15 s and then placed out of the pool. The spatial navigation test was performed on the 6th day to evaluate memory. In brief, the platform was removed, rats were placed in a random quadrant and the swimming trajectory was recorded within 90s. In the test, the proportion of swimming distance in the platform quadrant to the total swimming distance and the times of crossing the platform were calculated. The swimming distance in the platform quadrant reflects spatial localization and the times of crossing the platform reflects the accuracy of spatial memory. Before training, the platform was visible above the water surface, which may exclude rats with visual defects that were unable to find the platform. In addition, rats with poor performance in the test, such as those could not find the hidden platform and swam along the wall, were also excluded from this study.

**Fig. 1:**
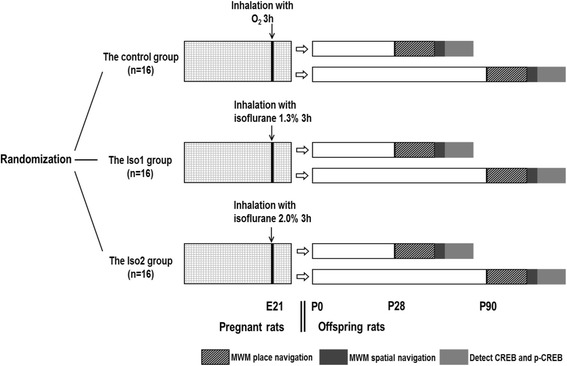
Study protocol. The E21 pregnant rats were randomized to inhalation with isoflurane 1.3%, 2.0% or O_2_. After the pregnant rats gave birth, the male offspring rats were randomized to day 28 after birth (P28) and day 90 after birth (P90), followed by MWM (place navigation 5 days and spatial navigation on the 6th day). At 2 h after the spatial navigation of MWM, detect the protein expression of CREB and p-CREB in the hippocampus

Two hours after the spatial navigation test, rats were intraperitoneally anesthetized with pentobarbital sodium. Half of each group of the rats were used to collect brain and followed by the separation of hippocampus. The hippocampus was weighed and lysed for total protein extraction. Samples were then stored at −80 °C for later use. Western blotting was performed to detect the protein expression of CREB and p-CREB in the hippocampus. The half of the rats were transcardially perfused with 4% paraformaldehyde and the brain was collected and fixed in 4% paraformaldehyde. Immunohistochemistry was performed to detect CREB and p-CREB expression. (Fig. 1).

The neonatal rats were randomly assigned into different groups to reduce variation. We normalized CREB and p-CREB protein expression in control group as 1. CREB and p-CREB expression in the Iso1 and Iso2 group was compared with the controls. All data are expressed as mean ± standard deviation. Statistical analyses were performed by using SPSS software (version 21.0; IBM, Corp., Armonk, NY, USA). One-Way ANOVA was used to compare the means between groups. A value of *P* < 0.05 indicated significance.

## Results

A total of 48 pregnant rats were used in this study, and eventually 316 male neonatal rats were used in the subsequent experiments. There were 52, 51 and 54 rats at day 28 after birth in the control, Iso1 and Iso2 group, respectively; there were 54, 51 and 54 rats at day 90 after birth in the control, Iso1 and Iso2 group, respectively (Table 1).

**Table 1:** The male offsprings in each group

	Control	Iso1	Iso2
P28	53-1^a^	51	54
P90	54	51	54
Total	107-1^a^	102	108

^a^1 rat in the control group was excluded due to poor performance in MWM test

**Fig. 2:**
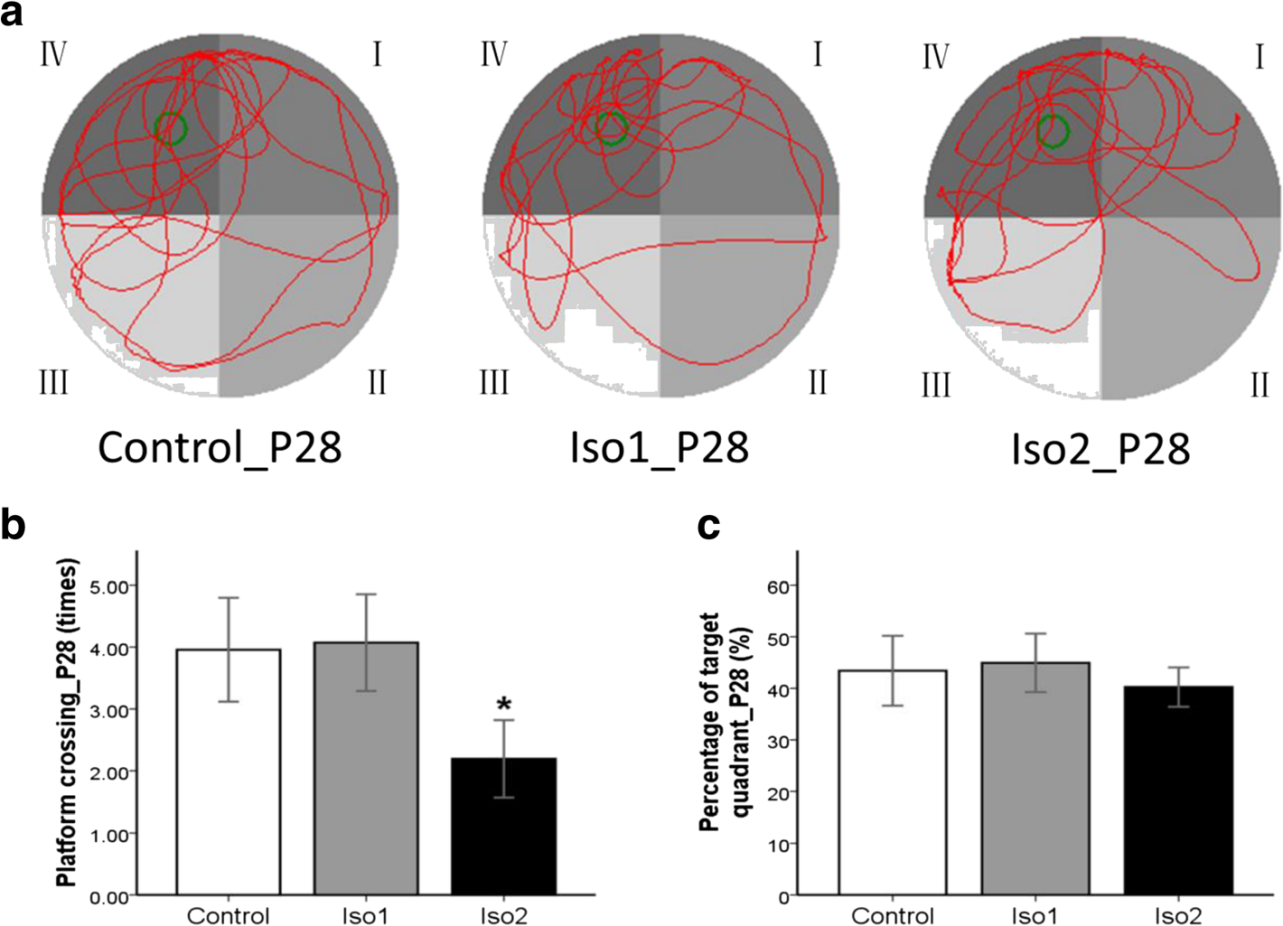
The track of MWM space exploration experiment at P28 of offsprings. **a**: The green circle in the diagram is the platform, the red line is the trajectories of rats. **b**: The times of crossing the platform. **c**: The percentage of platform quadrant. *: *P* < 0.05 vs. Control

**Fig. 3:**
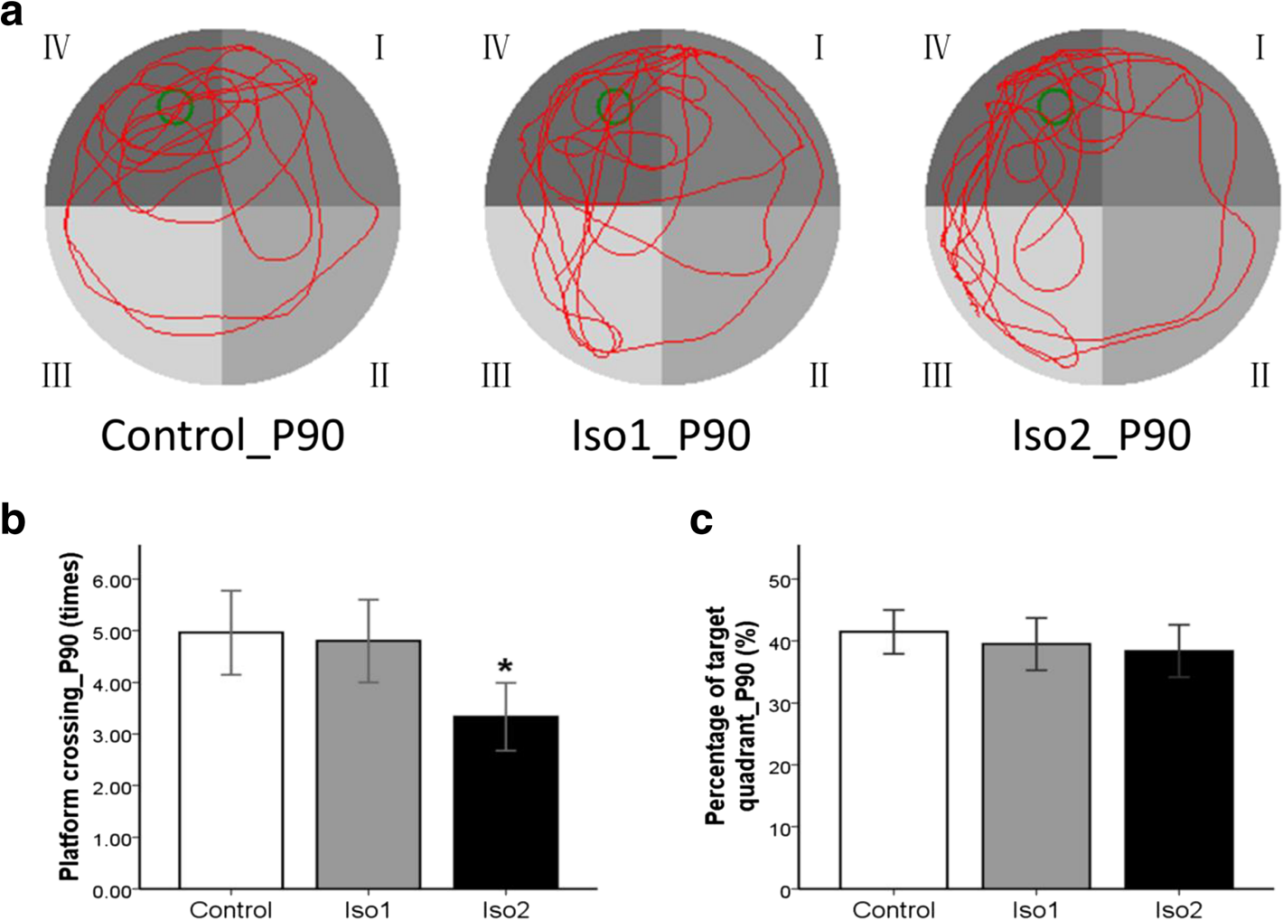
The track of MWM space exploration experiment at P90 of offsprings. **a**: The green circle in the diagram is the platform, the red line is the trajectories of rats. **b**: The times of crossing the platform. **c**: The percentage of platform quadrant. *: *P* < 0.05 vs. Control

There was no significant difference in the percentage of swimming distance in platform quadrant (IV quadrant) among three groups (*P* > 0.05). The times of crossing the platform in the Iso2 group was significantly lower than in the control group (*P* < 0.05) (Figs. 2 and 3).

**Fig. 4:**
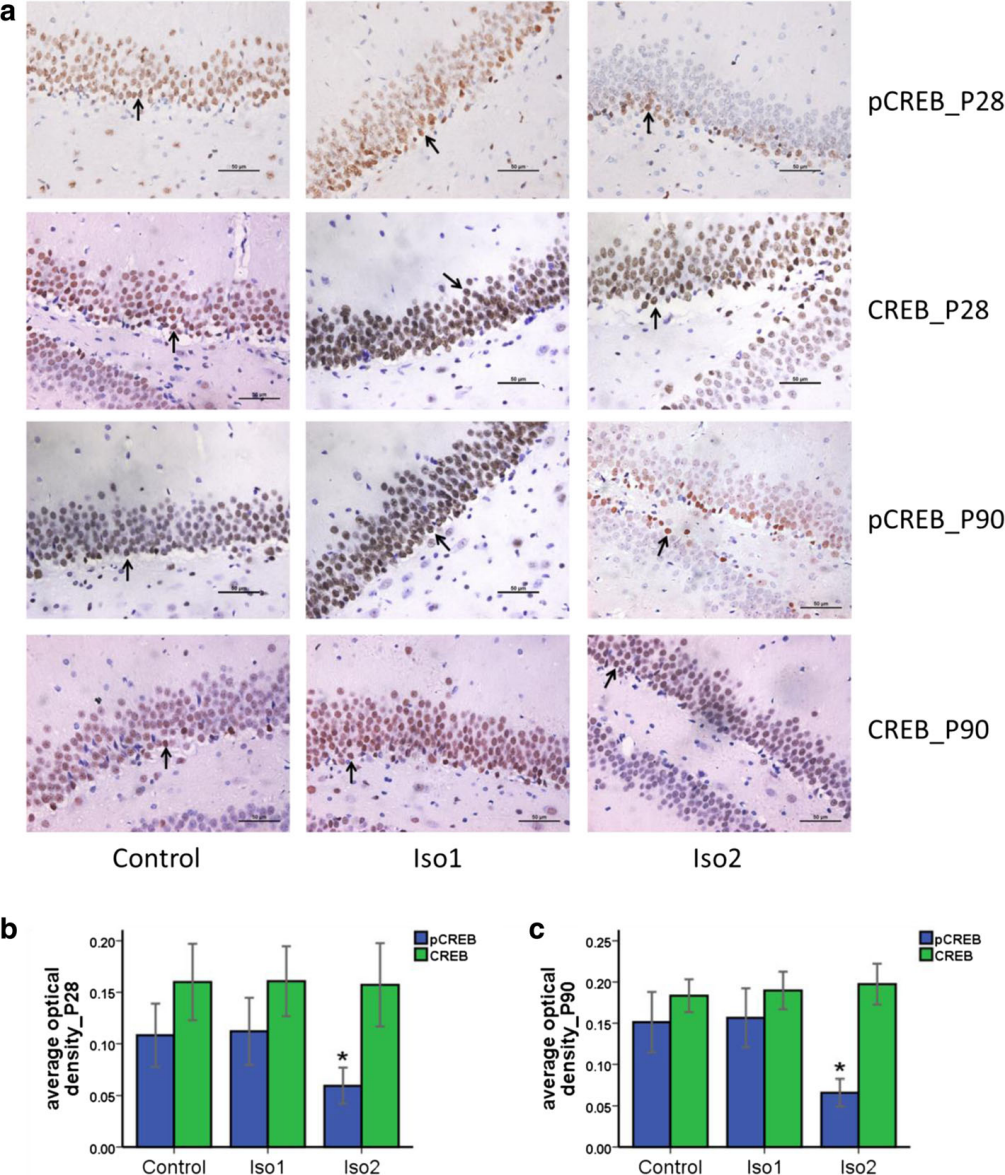
CREB and p-CREB expression in the granule cell layer of the hippocampus dentate gyrus of offspring rats. **a**: Arrow points positive cells. **b**: The P28 offspring rats. **c**: The P90 offspring rats. *: *P* < 0.05 vs. Control

CREB expression in the granule cell layer of the hippocampus dentate gyrus was comparable among the three groups (*P* > 0.05). p-CREB expression was mainly found in the whole granule cell layer of the hippocampus dentate gyrus in the control and Iso1 group, but mainly found in subgranular zone (SGZ) in the Iso2 group. In addition, p-CREB expression in the Iso2 group was significantly lower than in the Iso1 and control group (Fig. 4).

**Fig. 5:**
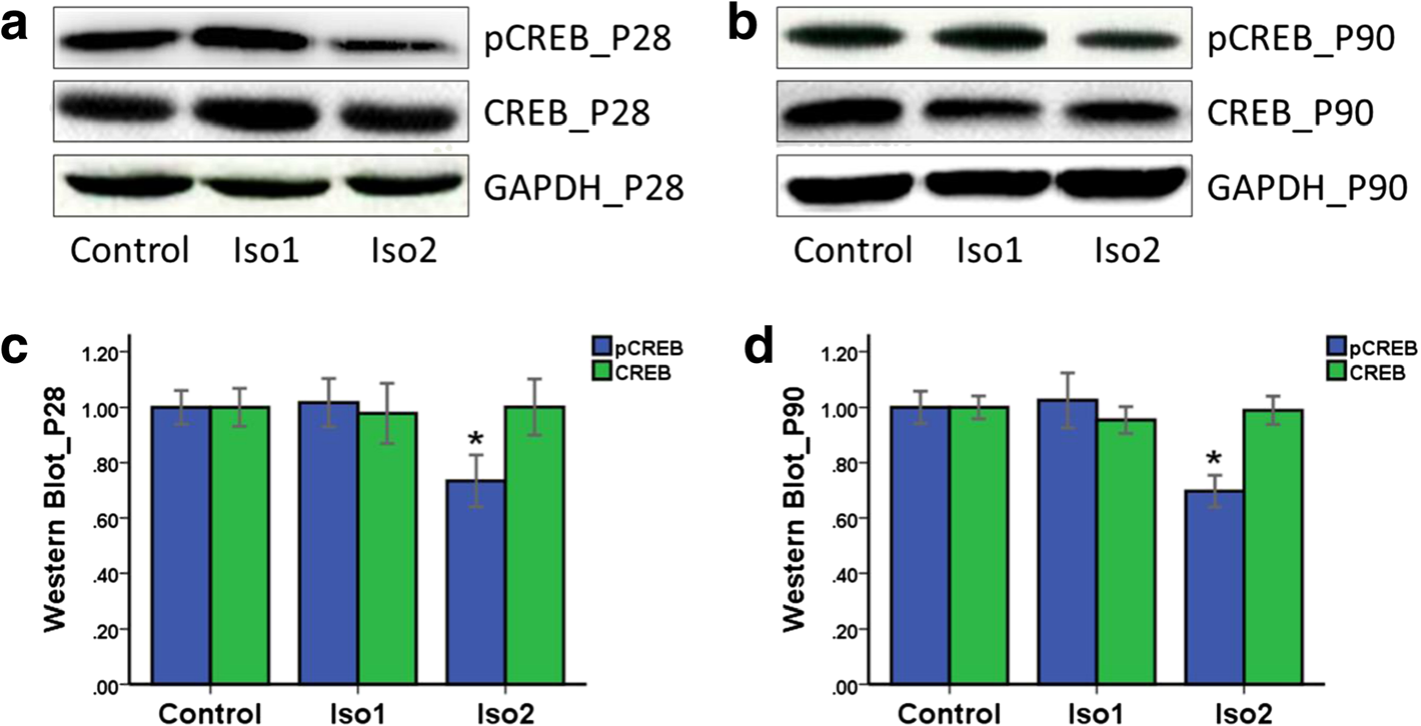
Western blotting of CREB and p-CREB expression in the hippocampus dentate gyrus of offspring rats. **a** and **c**: The P28 offspring rats. **b** and **d**: The P90 offspring rats. *: *P* < 0.05 vs. Control

CREB expression was similar among the three groups (*P* > 0.05). In addition, there was no marked difference in p-CREB expression between the Iso1 and control group (*P* > 0.05), however, p-CREB expression in the Iso2 group was significantly lower than in the control group (*P* < 0.05) (Fig. 5).

## Discussion

The growth and development of central nervous system are very complex in mammals. A substantial proportion of neurons undergo apoptosis during normal development. In synaptic plasticity phase, the nervous system is extremely sensitive to the internal and external environments and neurons that don’t form synapses will undergo apoptosis [3, 4]. Human brain development occurs mainly in fetus and mature slowly after birth [10], which is different from other species. For example, the nervous system of small rodents is largely immature at birth and rapidly developed after birth. Thus, in an animal study, brain development should be temporally equivalent to that in humans. It has been shown that brain development of rats at E21 is equivalent to that of human fetus at the gestational age of 12–16 weeks the second trimester [10, 11]. In this study, pregnant SD rats at E21 were exposed to isoflurane to mimic anesthesia on pregnant woman in the second trimester; isoflurane at 1.3% and 2.0% is equivalent to 1 and 1.5 MAC, respectively [12, 13].

The behavior MWM test is often employed as an effective tool to evaluate spatial learning and memory of rodents [14, 15]. In the spatial navigation test, the ratio of swimming distance in the platform quadrant to the total swimming distance reflects the capability of spatial location, and the times of crossing the platform reflects the accuracy of spatial memory. Our results showed that the ratio of swimming distance was comparable in the young (28 days) and adult (90 days) rats among the three groups, but the times of crossing the platform in the Iso2 group was significantly less than in the other two groups, indicating that isoflurane at a high concentration compromises the accuracy of spatial memory in rats but has little influence on their spatial localization.

The hippocampus is crucial to learning and memory [16–18]. The dentate gyrus in hippocampus is responsible for cognition and location navigation and transduces signals from the inner olfactory cortex to other regions of the hippocampus [19–21]. CREB is an important nuclear protein expressed widely in the cortex and hippocampus of adult rats. The dentate gyrus has the highest expression of CREB in the hippocampus [22]. CREB plays important roles in neurogenesis, synaptic formation, learning and memory [23, 24]; it regulates the transcription of a large number of genes, such as brain derived neurotrophic factor, c-fos, synaptic I and Ca/calmodulin-dependent protein kinases kinases or CaM kinases [25, 26], to form new synapses and gain long-term memory. Increased CREB expression and/or activity promotes memory formation [27, 28], and reduces CREB expression and/or activity inhibits memory formation [29–31]. Phosphorylated CREB was detected in cortical neurons with plasticity formation and hippocampal neurons after long-term potentiation stimulation and neurobehavioral training [32]. In addition, injection of CREB at dorsal hippocampus in mice was found to improve spatial memory in water maze test, but injection of the CREB variant that was unable to be phosphorylated at ser133 deteriorated spatial memory of these mice [33]. Increased p-CREB enhanced memory and cognitive abilities in mice [34]. Therefore, CREB phosphorylation contributes to the formation of memory. In this study, our results showed that p-CREB expression in the Iso2 group was significantly lower than in the control group and Iso1 group, which is consistent with the findings from MWM test. These findings confirm the crucial role of CREB phosphorylation in the formation of memory [33].

The cortex at the hippocampus dentate gyrus can be divided into the molecular layer, granular cell layer and polymorphic cell layer. Immunohistochemistry showed that CREB was expressed mainly in the granule cell layer of the dentate gyrus in offspring rats. In the control group and Iso1 group, p-CREB was expressed in the whole granule cell layer of the dentate gyrus, but its expression was only detectable in the subgranular zone (SGZ) in the Iso2 group. In 1998, Eriksson et al. confirmed neurogenesis in the dentate gyrus of humans for the first time [35]. Since then, increasing evidence has indicated a neural stem cell region in mammalian brain that is localized between the granular cell layer and hilus region with a size of 50–100 μm [36]. The region is also known as the subgranular zone. Neurons in the SGZ may differentiate into mature granular cells, some intermediate neurons, and glial cells, which are finally integrated into the granular cell layer [37–39]. These cells then form synapses, playing important roles in learning and memory. In the present study, the results showed that p-CREB was mainly expressed in SGZ of the dentate gyrus in the Iso2 group. Our results indicates that inhalation of isoflurane at a high concentration affects CREB phosphorylation in fetal brain without altering the CREB expression, which leads to compromised learning and memory. The new neural stem cells in SGZ in adulthood are not affected by the anesthetic and may further differentiate into granular cells and join the granular cell layer. Thus, the expression of CREB and p-CREB in SGZ remained unchanged. However, we could not exclude that isoflurane inhalation during pregnancy has little influence on neural stem cells in SGZ.

In the present study, pregnant rats were exposed to isoflurane for 3 h, which is equivalent to 48 h general anesthesia in humans. Other harmful stimulation was not employed aiming to reduce other confounding factors. However, in clinical practice, it is rare that pregnant women received anesthesia without surgery or surgery is performed under anesthesia for several weeks. Thus, although our results indicate that isoflurane has influence on neural development, we usually will not expect the equivalent conditions in general clinical practice.

## Conclusion

Inhalation of isoflurane at 1.3% during pregnancy has no significant influence on learning and memory of the offspring in rats; exposure to isoflurane at 2.0% during pregnancy affects the accuracy of spatial memory of the offspring, but has little influence on spatial localization, which is associated to inhibition of CREB phosphorylation in the granular cell layer of the dentate gyrus in the hippocampus of fetal rats.

## Abbreviations

CREB: CAMP-response element binding protein
E21: Gestational age of 21 days
MWM: Morris water maze
P28: Day 28 after birth
P90: Day 90 after birth
p-CREB: Phosphorylated cAMP-response element binding protein
SD pregnant rats: SpragueDawley pregnant rats
SGZ: Subgranular zone
